# Eye health risks associated with unclean fuel: a meta-analysis and systematic review

**DOI:** 10.3389/fpubh.2024.1434611

**Published:** 2025-01-30

**Authors:** Shi-Hang Chen, Yuan Tang, Song Xue

**Affiliations:** ^1^National Clinical Research Center for Metabolic Diseases, Metabolic Syndrome Research Center, Key Laboratory of Diabetes Immunology, Ministry of Education, and Department of Metabolism and Endocrinology, The Second Xiangya Hospital of Central South University, Changsha, China; ^2^Affiliated Hospital of Jiangxi University of Traditional Chinese Medicine, Jiangxi Provincial Key Research Laboratory of Traditional Chinese Medicine, Key Laboratory of Chronic Renal Failure, Nanchang, China

**Keywords:** health inequalities, cataracts, visual impairments, ocular symptoms, unclean cooking fuel

## Abstract

**Purpose:**

This study comprehensively examined the correlation between unclean cooking fuels (UCF) and ocular health, covering diverse eye conditions such as cataracts, visual impairments, and ocular discomfort.

**Methods:**

According to MOOSE and PRISMA guidelines, a meta-analysis and systematic review was conducted on 28 studies from 3 databases. Literature quality was assessed using the Newcastle-Ottawa Scale. Heterogeneity among articles was gaged with the *I*^2^ statistic, sensitivity analysis used ‘leave-one-out test’, and publication bias was evaluated using Egger, Begg tests, and funnel plot analysis.

**Results:**

The study evidenced a significant association between UCF exposure and cataracts [OR 2.29, 95% CI (1.24, 4.23)], visual impairments [OR 1.70, 95% CI (1.45, 2.00)], and eye diseases/symptoms [OR 2.03, 95% CI (1.25, 3.29)]. However, no correlation was found between UCF exposure and glaucoma or elevated intraocular pressure [OR 0.96, 95% CI (0.84, 1.10), *n* = 2]. Subgroup analysis revealed that UCF cooking had an impact on nuclear cataracts [OR 1.98, 95% CI (1.67, 2.33), *n* = 4]. But not on cortical cataracts [OR 1.25, 95% CI (0.98, 1.60), *n* = 3]. Additionally, UCF exposure was linked to severe visual impairments like night blindness [OR 2.03, 95% CI (1.00, 4.96)], blindness [OR 1.43, 95% CI (1.32, 1.55)], and specific ocular symptoms such as tearing while cooking (OR = 3.20), eye irritation (OR = 2.58), and red eyes (OR = 2.03).

**Conclusion:**

UCF cooking had significant impact on ocular health, notably on eye symptoms, cataracts, and visual impairments. UCF exposure presented demographic inequalities in cataract prevalence, while eye symptoms can serve as a reliable self-assessment of UCF exposure.

## Introduction

Visual impairment was one of the major barriers to unleashing human potential, significantly reducing productivity and educational attainment (1). In 2020, the prevalence of visual impairment reached 15,841 per 100,000 (with a range of 12,790.88 to 19,596.32) (2), resulting in a global productivity loss of up to $410.7 billion (1). Cataracts were significant components of visual impairment. According to the Global Burden of Disease (GBD) study, cataracts caused by household air pollution (HAP) accounted for 29.81% of the global disease burden, increasing to 50.72% in low-income regions (2), highlighting substantial health inequalities. The primary cause of HAP was the burning of unclean fuels (UCF), with approximately 2.6 billion people worldwide using these fuels for cooking (3). In 2019, nearly 2.3 million premature deaths were attributed to HAP (4), and the use of such fuels was notably higher in low-income areas compared to other regions (2). Therefore, for low-income populations, the preventive measure of improving fuel quality and cooking appliances may be more cost-effective compared to relying on cataract surgery after its onset.

Research indicated that the UCF exposure could lead to eye symptoms (5), including eye pain, blurred vision, redness, and tear while cooking (TWC), diminishing quality of life. Although these relatively common eye symptoms had not received as much research attention as visual impairment, they played an important role in the early indication of vision problems (6), and could serve as key marker for identifying specific populations at higher health risk due to HAP, especially after changes in cooking environments. Additionally, air pollution affected various chronic diseases (7–9), and using eye health as an assessment criterion might help raise awareness about UCF exposure.

The impact of UCF exposure on eye health has often been neglected by researchers in the past (10, 11). Moreover, the existing clinical studies did not comprehensively cover the types of fuels (12, 13) and eye health burden (14, 15), resulting in a lack of thorough assessment. Additionally, the conclusions drawn from different studies were contradictory (14, 16), highlighting the urgent need for an evidence-based medicine to claim the potential hazards of UCF on eye health.

Therefore, we conducted this systematic review and meta-analysis. Our study encompassed cataracts, visual impairments, ocular discomfort, glaucoma, and conjunctival diseases, while also performing subgroup analyses on fuel types, cataract subtypes, demographic characteristics. The significance of this research was identifying gaps in the current studies, exploring evidence related to health inequalities, and establishing effective health evaluation indicators.

## Methods

Although this meta-analysis was not formally registered, we diligently adhered to the Meta-analysis Of Observational Studies in Epidemiology (MOOSE) guidelines (17) and the Preferred Reporting Items for Systematic and Meta-analysis Protocols (PRISMA) guidelines (18) throughout the entire process.

### Search methods and inclusion/exclusion criteria

We searched all literature in the PubMed, Embase and Web of Science databases from their inception until November 10, 2023. Supplementary Table S1 provided an extensive list of detailed search terms and comprehensive information on the search strategy used in this study. No requirements were set for journal type. Studies must be published as original articles in English, encompassing clinical research types such as case–control studies, cross-sectional studies, cohort studies, case series studies, and randomized controlled trials. Conference abstracts, letters, books, review will be excluded. Additionally, studies lacking full-text access or available data will be excluded. All documents were imported into Endnote 20.

### Fuel categories and eye health metrics

UCF encompassed biomass fuels, solid fuels, wood, crop residue, coal, animal dung, kerosene, straw and charcoal. Clean fuel consisted of natural gas, biogas, liquefied petroleum gas (LPG), electricity, and propane. Ocular outcomes under study included cataracts, visual impairments, myopia, hyperopia, blindness, night blindness, eye discomfort, tearing while cooking (TWC), eye irritation and red eyes.

### Study selection and data extraction

**Figure 1:**
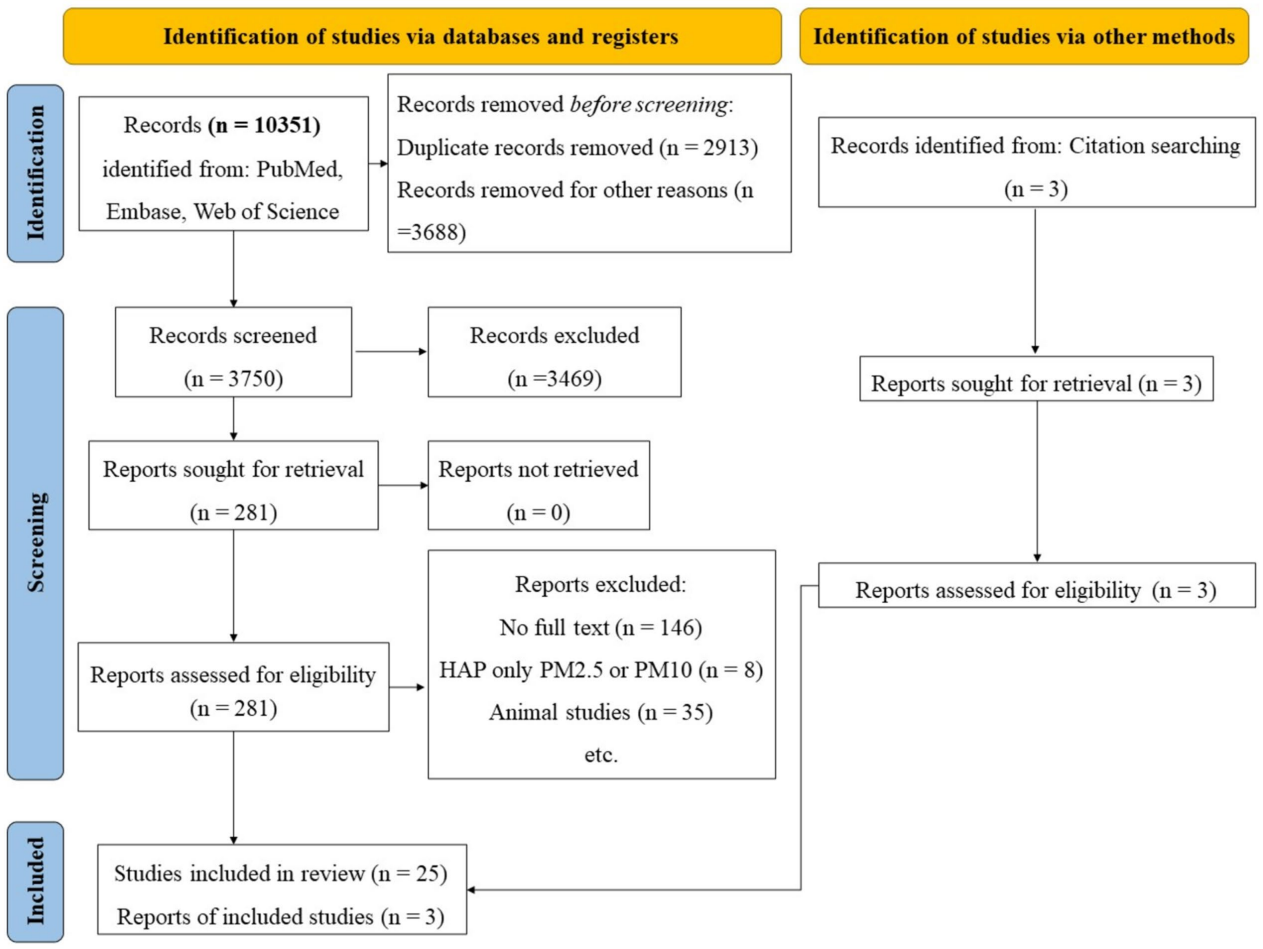
Flowchart of literature inclusion in the meta-analysis. HAP, Household air pollutants; PM2.5, Particulate matter 2.5.

Using Endnote X20 software, duplicate and unqualified research types of articles were removed. Two reviewers (S.C and Y.T) independently reviewed the remaining articles and excluded some articles that were obviously irrelevant to the research subject or animal experiment articles by title and abstract. For potentially relevant articles, the reviewers confirmed the articles that could be included in the study by intensively reading the full text. Finally, according to whether the research data could be converted into Odds ratio (OR), the literature was divided into included group or excluded group. Specific steps were shown in Figure 1. For ambiguous articles, the decision of whether to include the article was made after discussion with a third person (S.X). The original data of the pictures in the article through WebPlotDigitizer 4.5 software (19). The data extracted by this software will be specifically labeled in the figures.

### Search process and results

**Figure 2:**
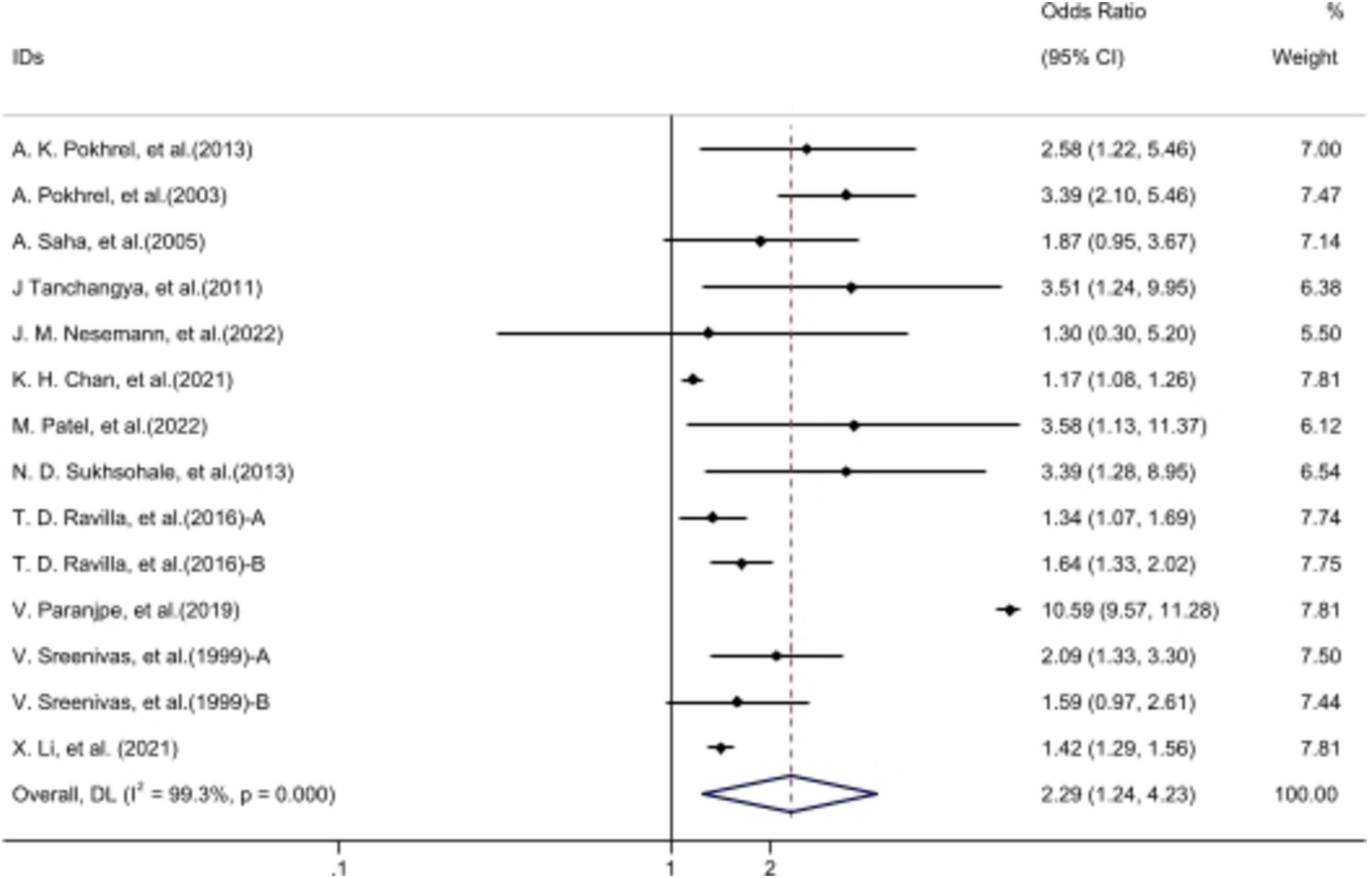
The forest plot: Comparison of cataracts in individuals using UCF vs. clean fuels. Ravilla et al. (15): A, male exposed to UCF/CF; B, female exposed to UCF/CF. Sreenivas et al. (14): A, data from Angamally, India; B, data from Calcutta, India. The data of ‘Patel et al. (5)’ from WebPlotDigitizer 4.5 software. Volunteers cooking with UCF exhibited a higher incidence of cataracts compared to those using clean fuels.

We conducted a search in the PubMed, Embase, and Web of Science databases, yielding a total of 10,351 articles. Among these, 2,913 articles were duplicate records, and 3,688 articles were excluded due to the absence of abstracts or inapposite article types. Ultimately, 3,750 articles underwent preliminary analysis. After carefully reviewing the titles and abstracts, we excluded 3,469 articles that were clearly unrelated to our study, leaving 281 articles for full-text review. Due to a lack of available original data or animal studies, we subsequently excluded 256 articles. In the end, a total of 25 articles were included in this study. Additionally, while reviewing the references of these 25 articles, we identified another three relevant articles that had not been previously included, and we added them to the analysis (Figure 2).

### Statistical analysis

**Figure 3:**
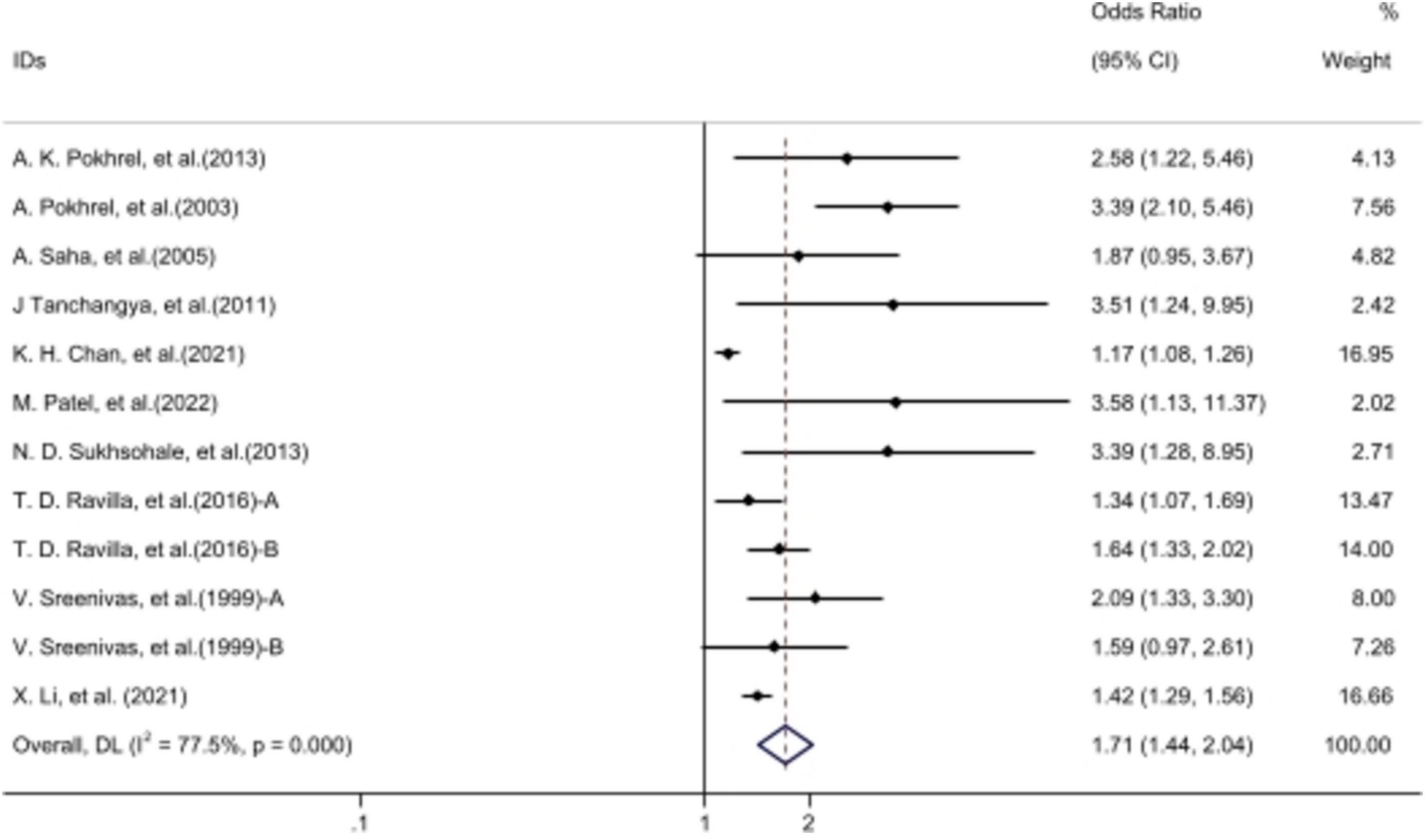
The forest plot: comparison of cataracts in individuals using UCF vs. clean fuels after revision. Ravilla et al. (15): A, male exposed to UCF/CF; B, female exposed to UCF/CF. Sreenivas et al. (14): A, data from Angamally, India; B, data from Calcutta, India. The data of ‘Patel et al. (5)’ from WebPlotDigitizer 4.5 software. Nesemann et al. (52), Paranjpe et al. (12) were excluded. Volunteers cooking with UCF exhibited a higher incidence of cataracts compared to those using clean fuels after revision.

We conducted a comprehensive systematic review on UCF exposure and eye health, and performed a meta-analysis on studies with two or more articles. All analyses were performed using Excel 2017, Stata/MP (version 17), and Adobe Illustrator 2018 for data processing, statistical computations, and image generation, respectively. Forest plots were utilized to depict the adverse effects of UCF on ocular health. Data were aggregated to calculate OR values alongside 95% confidence intervals (CI). Outcomes from the included studies were assessed using either random or fixed effects models, chosen based on the degree of heterogeneity determined by the I-squared (*I*^2^) statistic. If *I*^2^ > 50%, a random effects model was utilized for meta-analysis; otherwise, a fixed effects model was applied (20). Sensitivity analysis involved a systematic investigation of each article’s impact on the outcomes (21). Publication bias was evaluated using both Egger’s test and Begg’s suggestion (22). A significance threshold of *p*-value (*p*) < 0.05 was set for all analytical outcomes (Figure 3; Table 1).

**Table 1:** The basic information of included articles.

Author	Year	Country	Study type	NOS scores
Ellegård et al. (43)	1997	Zambia, Mozambique, Vietnam	Cross-sectional study	7
Pokhrel et al. (13)	2013	Nepal	Cross-sectional study	7
Pokhrel et al. (44)	2005	India	Cross-sectional study	6
Saha et al. (45)	2005	India	Cross-sectional study	6
James et al. (46)	2020	India	Cross-sectional study	4
Norbäck et al. (47)	2019	China	Multicenter study	6
Walker, et al. (48)	2020	Honduras	Cohort study	4
Diaz et al. (49)	2007	Guatemalan	Cohort study	7
IDas et al. (50)	2017	Malawi	Cross-sectional study	4
Tanchangya et al. (16)	2011	Bangladesh	Case–control study	5
Katz et al. (51)	2009	India	Case–control study	5
Nesemann et al. (52)	2022	India	Cohort study	7
Raufman et al. (53)	2020	Kenya	Case–control study	4
Sahoo et al. (54)	2023	India	Case–control study	4
Chan et al. (37)	2021	China	Case–control study	6
Zheng et al. (55)	2016	Peru	Cohort study	7
Patel et al. (5)	2022	Nepal	Cohort study	5
Sukhsohale et al. (56)	2013	India	Cross-sectional study	4
Adhikari et al. (57)	2018	Nepal	Case–control study	7
Islam et al. (58)	2022	India	Cross-sectional study	6
Ravilla et al. (15)	2016	India	Cross-sectional study	6
Aung et al. (59)	2018	India	Cohort study	7
Paranjpe et al. (12)	2019	India	Case–control study	3
Sreenivas et al. (14)	1999	India	Case–control study	5
Mishra et al. (60)	2001	India	Cross-sectional study	7
Kushk et al. (61)	2005	Pakistan	Cohort study	4
Li et al. (62)	2021	China, India, Mexico, Russia, South Africa, Ghana	Cross-sectional study	7
Zhou et al. (63)	2023	China	Cohort study	7

NOS, Newcastle-Ottawa Scale.

## Outcomes

### Study characteristics

All 28 studies were conducted in developing countries, with 14 taking place in India, with a few carried out in Latin America and Africa. As for the study types, 11 cross-sectional studies, 8 cohort studies, 8 case–control studies, and 1 multicenter study were included. The literature was evaluated using the NOS (23), with scores ranging between 3 to 7 across all articles (Table 2). In terms of subject matter, 14 articles focused on cataracts, 8 studies addressed visual impairments, and 10 articles discussed ocular symptoms. In contrast, there was only one article that explored topics such as glaucoma, conjunctivitis, and night blindness. Supplementary Figure S1 presented a summary of the forest plots in this meta-analysis; Supplementary Figure S2 provided an overview of the funnel plots in this meta-analysis; Supplementary material S1 outlined the sensitivity analysis results of this meta-analysis.

**Table 2:** The meta-analysis outcomes: comparison of eye healthy problems in individuals using UCF vs. clean fuels.

Eye outcomes	Cooking fuel (UCF/ Clean fuel)			*p* of Publication bias	
	No. of study	OR (95%CI)	I^2^ (%)	Egger test	Begg test
Vision loss or impairment					
Visual impairment	8	1.70 (1.45, 2.00)	75.8	0.044	0.266
Myopia	2	1.44 (1.39 1.49)	0.0	–	–
Hyperopia	2	1.34 (1.08, 1.66)	95.8	–	–
Blindness	5	1.43 (1.32, 1.55)	45.6	0.777	1.000
Night blindness	1	2.03 (1.00, 4.96)	–	–	–
Eye diseases or uncomfortable					
TWC	4	3.20 (2.45, 4.19)	34.6	0.864	0.734
Eye irritation	10	2.58 (1.82, 3.66)	72.0	0.574	0.474
Red eyes	4	3.81 (1.73, 7.67)	0.0	0.266	0.734
Other symptoms	5	2.03 (1.25, 3.29)	68.2	0.555	0.806
Cataract					
All	14	2.29 (1.24, 4.23)	99.3	0.830	0.228
Nuclear Cataract	4	1.98 (1.67, 2.33)	11.5	0.528	0.734
Cortical Cataract	3	1.25 (0.98, 1.60)	0.0	0.074	0.296
Glaucoma or elevated IOP					
All	2	0.96 (0.84, 1.10)	0.0	–	–
Glaucoma	1	0.95 (0.82, 1.09)	–	–	–
Elevated IOP	1	1.14 (0.65, 1.99)	–	–	–
Conjunctival disease					
All	2	2.04 (0.83, 5.00)	90.8	–	–
Conjunctivitis	1	3.30 (2.05, 5.32)	–	–	–

TWC, Tear while cooking; IOP, Intraocular pressure.

### Unclean cooking fuels and cataract

Fourteen articles demonstrated a significant association between UCF and cataracts [OR 2.29, 95% CI (1.24, 4.23)]. Despite considerable heterogeneity among studies (*I*^2^ = 99.1%), sensitivity analysis indicated relatively stable outcomes (Supplementary material S1). Funnel plot analysis showed an even distribution of articles on both sides (Supplementary Figure S2), with *p* > 0.05 from publication bias tests (Table 2), indicating no such bias. After excluding two articles out of the 95% CI of funnel plot, exposure to biomass fuels still had a negative impact on the incidence of cataracts [OR 1.71 95% CI (1.44, 2.03)] and the heterogeneity dropped to 77.5%.

Among the five articles discussing the relationship between kerosene exposure and cataracts, there was low heterogeneity among them (*I*^2^ = 14.4%). Meta-analysis outcomes suggested a potential link between kerosene cooking and cataract development [OR 1.48, 95% CI (1.11, 1.97)] (Supplementary Figure S1), yet Egger’s analysis indicated potential publication bias (*p* = 0.03). The results of the subgroup analysis showed a significant association between UCF exposure and nuclear cataracts, while no significant correlation was found with cortical cataracts. Additionally, the impact of UCF exposure on females (OR 1.28) was greater than that on males (OR 1.23). For more results from the subgroup analysis, please refer to Table 2.

### Unclean cooking fuels and visual impairment

Meta-analysis revealed a positive association between UCF-based cooking and visual impairments [OR 1.70, 95% CI (1.45, 2.00)] (Supplementary Figure S1), with substantial heterogeneity among these studies (*I*^2^ = 75.8%). Sensitivity analysis showed that the meta-analysis results were stable, with the lowest 95% CI being 1.37 (Supplementary material S1). While the funnel plot exhibited symmetry, Egger’s analysis suggested potential publication bias (*p* = 0.04). Among other vision loss catalogs, five touch upon blindness [OR 1.43, 95% CI (1.32, 1.55)], two upon myopia [OR 1.44, 95% CI (1.39, 1.49)], two upon hyperopia [OR 1.34, 95% CI (1.08, 1.66)], and one specifically examined night blindness [OR 2.03 95% CI (1.00, 4.96)]. Following a comprehensive review, the findings were considered credible.

### Unclean cooking fuels and eye symptoms or other diseases

Eye symptoms were mostly self-assessed by patients and obtained through questionnaires. Therefore, we selected three most commonly used eye symptoms: (TWC, eye irritation, red eyes). Meta-analysis suggested that exposure to UCF wound promoted the development of these symptom (Table 2). The sensitivity analysis showed that the results were stable and there was no publication bias (Table 2). Only articles related to eye irritation symptoms had high heterogeneity (*I*^2^ = 72.0%). Five articles did not cite specific types of eye disease or symptom, so we combined them as a broad concept as other eye disease or symptom, and we found that exposure to UCF cooking increased the occurrence of this catalog [OR 2.03, 95% CI (1.25, 3.29)], but the heterogeneity between articles was high (*I*^2^ = 68.2). The sensitivity analysis indicated that the meta-analysis results were stable. Both Egger test and Begg test believed that the relevant studies had no publication bias (Table 2).

## Discussion

Our research indicated that UCF exposure was closely related to eye health. This association was supported by foundational studies, showing that the combustion of UCF had low efficiency (24, 25), generating a significant amount of particulate matter and gaseous pollutants (26), including carbon monoxide and nitrogen oxides (27, 28). These pollutants could directly cause eye inflammation (15) or indirectly affect eye health by increasing reactive oxygen species release (29, 30) and decreasing dopamine release (31).

Our study demonstrated that the health burden of cataracts associated with UCF exposure varied based on fuel type, country, cataract type, exposure duration, gender, fuel conversion, and urban–rural status. For instance, patients with over 40 years of cooking time had a higher incidence of cataracts (OR 1.16) compared to those with 1–19 years of cooking time (OR 1.09). Additionally, the impact of UCF cooking on women (OR 1.28) was greater than on men (OR 1.23) (32), likely because women were typically the primary cooks and had longer cooking durations. Patients transitioning from biomass fuels to clean fuels had lower odds ratios than those who continued using biomass fuels (33) (Table 3), suggesting that early switching to cleaner cooking fuels might help mitigate health risks.

**Table 3:** The meta-analysis and subgroup analysis outcomes: comparison of cataract in individuals using UCF vs. clean fuels.

Subgroup	Cooking fuel (UCF/ clean fuel)			*p* of publication bias	
	No. of study	OR (95%CI)	*I*^2^ (%)	Egger test	Begg test
Fuel type					
Wood	4	1.17 (1.11, 1.23)	64.1	0.017	0.308
Kerosene	6	1.48 (1.11, 1.97)	14.4	0.028	0.452
Straw	1	2.86 (1.10, 7.45)	–	–	–
Coal	1	1.17 (1.09, 1.24)	–	–	–
Dung	1	0.46 (0.21, 1.00)	–	-	–
Country					
China	2	1.23 (1.15, 1.32)	88.6	–	–
India	8	2.28 (0.92,5.64)	98.7	0.073	0.386
Nepal	2	2.12 (1.23, 3.65)	73.7	–	–
Bangladesh	1	3.51 (1.24, 9.95)	–	–	–
Mexico	1	1.23 (0.84, 1.81)	–	–	–
Russia	1	1.52 (0.96, 2.38)	–	–	–
South Africa	1	0.88 (0.62, 1.24)	–	–	–
Ghana	1	0.92 (0.50, 1.71)	–	–	–
Type of cataract					
Nuclear Cataract	4	1.98 (1.67, 2.33)	11.5	0.528	0.734
Cortical Cataract	3	1.25 (0.98, 1.60)	0.0	0.074	0.296
Cooking year					
1–19	3	1.09 (1.01, 1.18)	0.0	0.079	0.296
20–39	4	2.16 (2.07, 2.25)	99.8	0.770	0.734
>40	3	1.16 (1.10, 1.22)	0.0	0.624	0.602
Gender					
Male	4	1.23 (1.03, 1.49)	78.3	0.120	0.734
Female	6	1.28 (1.20, 1.36)	90.3	0.056	0.805
Fuel type conversion					
Always Clean	1	1.00 (0.95, 1.05)	–	–	–
Biomass to Clean	3	1.05 (1.01, 1.09)	0.0	0.274	0.602
Always Biomass	2	1.18 (1.09, 1.41)	71.8	–	–
Location					
Urban	1	1.24 (1.09, 1.41)	–	–	–
Rural	1	1.74 (1.51, 2.00)	–	–	–

Furthermore, based on a larger data source, our research confirmed that UCF exposure was significantly associated only with nuclear cataracts [OR 1.98, 95% CI (1.67, 2.33)], not with cortical cataracts [OR 1.25, 95% CI (0.98, 1.60)]. Sensitivity analyses yielded stable results (Supplementary material S1), with no evidence of heterogeneity (Table 3) or publication bias. It remains unclear whether nuclear cataracts are more sensitive to air pollution or the limited number of studies on cortical cataracts led to false negatives, indicating a need for further research to clarify this issue.

In low-income countries, the burden of cataracts was relatively high, and studies showed that cataract surgery was cost-effective (34). However, due to limited access to medical services, poor quality of care, and cultural beliefs, it was often challenging to reach those in need, even when financial resources were sufficient (35, 36). Therefore, we suggested that preventing exposure to UCF, enhancing health education, and providing targeted cataract surgeries could be more effective and economical strategies.

The limited number of studies examining the link between UCF exposure and some other eye conditions necessitates descriptive analysis only. Articles explored glaucoma (37) or increased intraocular pressure (IOP) (38), both indicating no significant relationship between UCF exposure and them [OR 0.96, 95% CI (0.84, 1.10)]. A study by the China Kadoorie Biobank (37), encompassing 512,715 adults aged 30 to 79 across 10 areas in China from 2004 to 2008, found that exposure to solid fuels positively correlated with an increased prevalence of Conjunctiva disorder [OR 1.32, 95% CI (1.25, 1.39), *n* = 4,877] and disorders affecting the sclera, cornea, iris, and ciliary body [OR1.37, 95% CI (1.22, 1.48), *n* = 1,583]. Similarly, a research observed higher biomass fuel exposure levels among patients with conjunctivitis (39). However, in order to gain a comprehensive understanding and confirm the reliability of these results, additional detailed studies were needed in the future.

Previous studies indicated that visual impairment exhibited significant health inequalities between different income countries (2). Specifically, the incidence of visual impairment in low-income countries was eight times higher than in high-income countries, while in middle-income countries, it was four times higher (1). This disparity might be linked to high exposure to UCF in low-income regions. As the cost of clean fuels (such as LPG) was significantly higher than that of UCF (40), local residents often found it unaffordable, resulting in unequal access to fuel (40). To bridge this gap, reducing income inequality was crucial. Policy improvements, such as promoting remittance inflows or providing subsidies for fossil fuels, could enhance the availability of clean cooking fuels (40, 41).

Our research showed that eye symptoms effectively reflected individuals’ exposure levels to UCF, supporting the scientific validity of UCF-related questionnaire designs. Therefore, it was essential to prioritize measures to reduce UCF exposure for patients exhibiting eye symptoms. For low-income groups with limited financial means who could not access to clean fuels, it was recommended to utilize open or well-ventilated cooking environments, or to wear protective eyewear to minimize direct contact between the eyes and smoke. For women with long-term UCF exposure and users with better economic conditions, we advised transitioning to clean fuels as soon as possible. Additionally, we recommended that patients experiencing significant eye symptoms during cooking undergo chronic disease screenings to identify potential health issues promptly (42).

### Limitation

This study was unable to access individual-level data, limiting analysis and summarization to the population level. Most studies included were retrospective, with few prospective studies, affecting the reliability of results. Self-assessment of eye symptoms by patients without medical examinations might introduce bias.

## Conclusion

UCF usage was significantly linked to eye health issues, notably eye symptoms, cataracts, and visual impairments. Further prospective and foundational research was crucial to authenticate potential impacts and underlying mechanisms, addressing data limitations and mitigating biases arising from self-assessment.
