# Supplementary material for: Eye health risks associated with unclean fuel: a meta-analysis and systematic review
Source: Front Public Health. 2025 Jan 30;12:1434611. doi: 10.3389/fpubh.2024.1434611 (PMC11821592; doi:10.3389/fpubh.2024.1434611)
Supplement: Supplementary file 1 [file Table_1.docx]

Supplemental Table 1: The search strategies and outcomes of this meta-analysis

| Ocular symptoms | Search query | Search results |
| --- | --- | --- |
| Cataract | (Cataract OR Clouding of the lens OR Opacity of the lens) AND (biomass OR solid fuel OR clean fuel OR household air pollution OR indoor air pollution OR pollution fuel OR cooking fuel OR heating fuel) | PubMed: 47  Embase: 71  Web of Science: 112 |
| Glaucoma | Glaucoma OR Increased pressure within the eye OR Elevated intraocular pressure OR Damage to the optic nerve due to glaucoma OR increased eye pressure OR Eye pressure) AND (biomass OR solid fuel OR clean fuel OR household air pollution OR indoor air pollution OR pollution fuel OR cooking fuel OR heating fuel) | PubMed: 32  Embase: 77  Web of Science: 173 |
| Disease of conjunctiva | (Disorders of conjunctiva OR Conjunctivitis OR Pterygium OR Conjunctival degenerations and deposits OR Conjunctival scars OR Conjunctival hemorrhage OR Conjunctivitis OR Dry Eye Syndrome OR Subconjunctival Hemorrhage OR Conjunctival Cyst OR Conjunctival Scarring OR Conjunctival Lesions OR Conjunctival disease) AND (biomass OR solid fuel OR clean fuel OR household air pollution OR indoor air pollution OR pollution fuel OR cooking fuel OR heating fuel) | PubMed: 103  Embase: 202  Web of Science: 231 |
| Scleral, corneal, iris, and ciliary body diseases | (Disorders of sclera OR Scleritis OR Episcleritis OR Keratitis OR Corneal ulcer OR Keratoconjunctivitis OR Corneal neovascularization OR Adherent leukoma OR Corneal scars and opacities OR cornea OR Iridocyclitis OR disorders of iris OR Sclera OR Scleritis OR Cornea OR Keratitis OR Iris OR Iritis OR Ciliary Body OR Cyclitis) AND (biomass OR solid fuel OR clean fuel OR household air pollution OR indoor air pollution OR pollution fuel OR cooking fuel OR heating fuel) | PubMed: 135  Embase: 372  Web of Science: 418 |
| Visual Impairment | (Visual Impairment OR Decreased Vision OR Diminished Eyesight OR Reduced Visual Acuity OR Loss of Vision OR Declining Eyesight OR Deteriorating Vision OR Refractive Error OR Vision Defect OR Focusing Problem OR Myopia OR Hyperopia OR Astigmatism OR Presbyopia OR Inaccurate Vision OR Visual disturbances OR blindness OR Amblyopia ex anopsia OR Diplopia OR Visual field defects OR Color vision deficiencies OR Night blindness OR visual impairment) AND (biomass OR solid fuel OR clean fuel OR household air pollution OR indoor air pollution OR pollution fuel OR cooking) | PubMed: 552  Embase: 294  Web of Science: 1064 |
| Eye uncomfortable | (Eye Health OR Eye Discomfort OR eye uncomfortable OR self-reported symptoms OR tear while cooking OR eye irritation OR red eyes or eye) AND (biomass OR solid fuel OR clean fuel OR household air pollution OR indoor air pollution OR pollution fuel OR cooking) | PubMed: 1186  Embase: 1350  Web of Science: 3621 |
| Cataract | (Kerosene OR Paraffin oil OR Lamp oil OR Coal oil) and (Cataract OR Clouding of the lens OR Opacity of the lens) | PubMed: 67  Embase: 180  Web of Science: 61 |

Supplemental Figure 1:


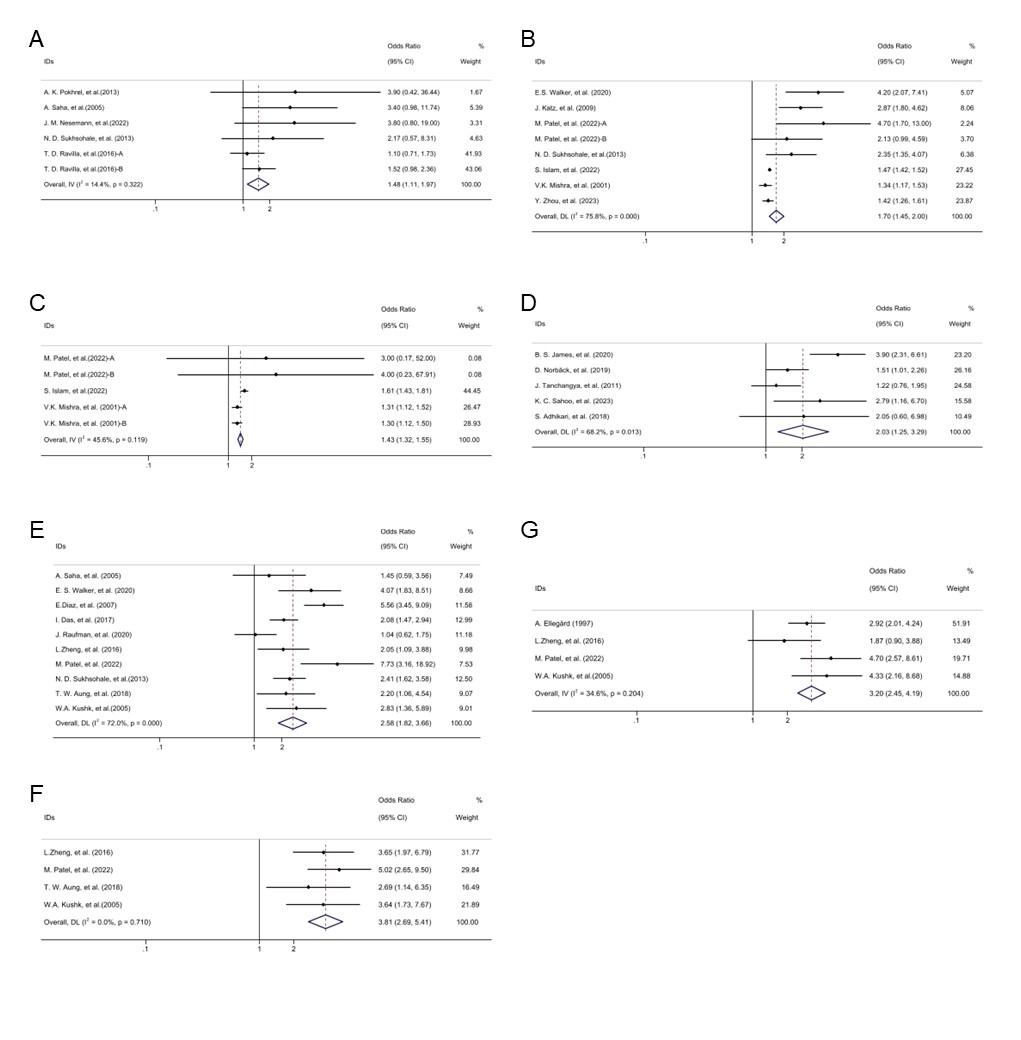


Supplemental Figure 1: A, the forest plot between kerosene cooking and cataract publication; B, the forest plot between unclean fuel cooking and visual impairment; C, the forest plot between unclean fuel cooking and blindness; D, the forest plot between unclean fuel cooking and eye disease and symptoms; E. the forest plot between unclean fuel cooking and eye irritation; G. the forest plot between unclean fuel cooking and tear while cooking symptoms; F, the forest plot between unclean fuel cooking and red eyes.

T. D. Ravilla, et al. (2016): A, male exposed to UCF/CF; B, female exposed to UCF/CF. M. Patel, et al. (2022): A. data from right eyes; B, data from left eyes. V.K. Mishra, et al. (2001): A, male exposed to UCF/CF; B, female exposed to UCF/CF.

​Meta-analysis outcomes revealed a higher likelihood of cataracts among individuals cooking with kerosene compared to clean fuels. Cooking with UCF showed a greater propensity for visual impairments, blindness, and eye tissue issues compared to clean fuels.

Supplemental Figure 2:


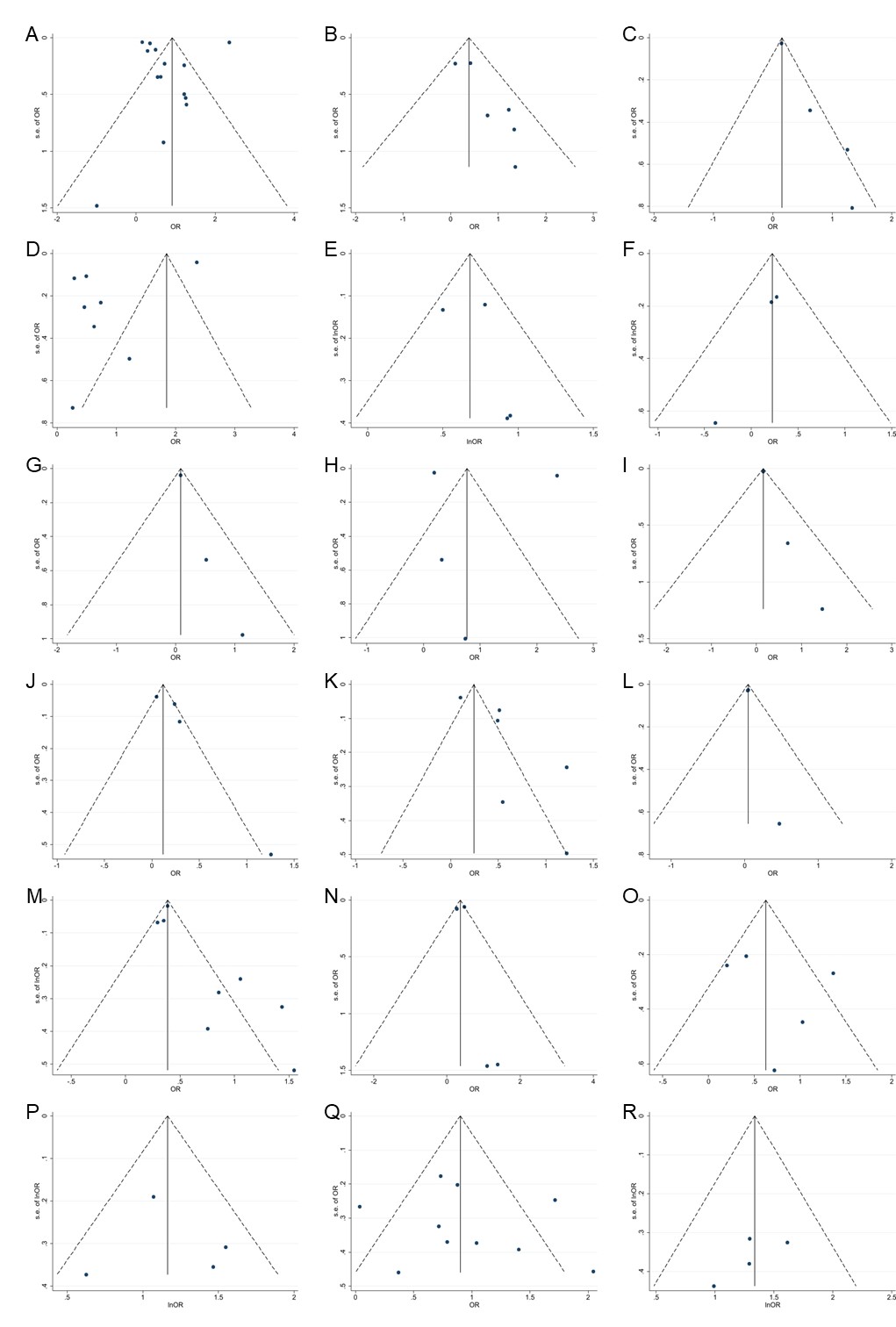


Supplemental Figure 2: The funnel plots between UCF and eye healthy problems

A, The funnel plots between UCF and cataract; B, The funnel plots between kerosene and cataracts; C, The funnel plots between wood fuel and cataract; D, The funnel plots between UCF and cataract in India; E, The funnel plots between UCF and nuclear cataract; F, The funnel plots between UCF and cortical cataract; G, Funnel Plot: Comparison of Cataracts in Individuals Using UCF for 1-19 Years vs. Clean Fuels; H, Funnel Plot: Comparison of Cataracts in Individuals Using UCF for 20-39 Years vs. Clean Fuels, I, Funnel Plot: Comparison of Cataracts in Individuals Using UCF for more than 40 Years vs. Clean Fuels; J, Funnel Plot: Comparison of Cataracts in Male Using UCF vs. Clean Fuels; K, Funnel Plot: Comparison of Cataracts in Female Using UCF vs. Clean Fuels; L, Funnel Plot: Comparison of Cataracts in Person Using UCF before but Using Clean Fuels Now vs. Always Using Clean Fuels; M, The funnel plots between UCF and visual impairment; N, The funnel plots between UCF and blindness; O, The funnel plots between UCF and eye disease or symptoms; P, The funnel plots between UCF and TWC; Q, The funnel plots between UCF and eye irritations; R, O, The funnel plots between UCF and red eyes
